# Incidence and determinants of hypophosphatemia in diabetic ketoacidosis: an observational study

**DOI:** 10.1136/bmjdrc-2020-002018

**Published:** 2021-02-17

**Authors:** Amarens van der Vaart, Femke Waanders, André P van Beek, Titia M Vriesendorp, B H R Wolffenbuttel, Peter R van Dijk

**Affiliations:** 1 Department of Endocrinology, University of Groningen, University Medical Center Groningen, Groningen, The Netherlands; 2 Department of Internal Medicine, Isala, Zwolle, The Netherlands

**Keywords:** diabetes mellitus, type 1, diabetic ketoacidosis

## Abstract

**Introduction:**

Diabetic ketoacidosis (DKA) is a life-threatening complication of type 1 diabetes mellitus (T1DM) characterized by hyperglycemia and metabolic acidosis. Hypophosphatemia in DKA often occurs during hospital admittance for DKA. Literature on the magnitude, determinants and consequences of hypophosphatemia in DKA is scarce. Primary aim of this study was to investigate the incidence and consequences of hypophosphatemia during hospitalisation for DKA.

**Research design and methods:**

Cohort study among individuals with T1DM who were admitted for DKA between 2005 and 2020 in an academic and a non-academic hospital. Multivariate regression models were performed to investigate determinants of the lowest phosphate during the treatment of DKA.

**Results:**

A total of 127 episodes of DKA among 80 individuals were identified. Age at DKA presentation was 28 (22–46) years, 45% of the cases was female, diabetes duration was 13.2 (8.9–25.5) years with glycosylated hemoglobin levels of 91.9±26.2 mmol/mol. In 9% of all cases, DKA was the first presentation of T1DM. Lowest phosphate levelss reported during the treatment phase were 0.54 (0.32–0.83) mmol/L and hypophosphatemia was present in 74% (62/84). The time to lowest phosphate was 16 (8–23) hours. In multivariate analysis, baseline bicarbonate and hemoglobin at admission were significantly associated with the lowest phosphate level reported. No adverse effects of hypophosphatemia on hospital stay duration, morbidity or mortality were found, even if left untreated.

**Conclusions:**

Hypophosphatemia during DKA is common and increases with severe acidosis. However, in this study it was not related to adverse outcomes. Although limitations of this retrospective study should be taken into account, the routine and repeated measurement of phosphate levels in DKA could be reconsidered, provided that possible symptoms related to hypophosphatemia are monitored.

**Significance of this study**

**What is already known about this subject?**

- In diabetic ketoacidosis (DKA), net (urinary) loss of phosphate occurs because of a transcellular shift, osmotic diuresis and reduced renal phosphate reabsorption.

**What are the new findings?**

- In this study, the incidence of hypophosphatemia during the treatment phase of DKA is 74%.
- The main determinant of hypophosphatemia is the severity of acidosis.
- We observed no negative clinical outcomes related to hypophosphatemia.

**How might these results change the focus of research or clinical practice?**

- The routine assessment and treatment of hypophosphatemia during a DKA may be reconsidered.

## Introduction

Diabetic ketoacidosis (DKA) is an acute and life-threatening complication of type 1 diabetes mellitus (T1DM), with an estimated mortality rate of DKA of 2%–14%. The incidence of DKA is 46–80 per 10 000 persons‐years.^1^ In most cases, DKA is caused by an infection, limited compliance to insulin therapy and—in approximately 20%—new-onset diabetes. DKA is characterized by insulin deficiency, causing hyperglycemia and high amounts of ketone bodies leading to acidosis. During DKA, the body uses ketone bodies as a surrogate source of energy.^2^ Both hyperglycemia and metabolic acidosis contribute to volume depletion and electrolyte deficiencies in the intracellular and extracellular environment. One of these electrolyte disturbances is hypophosphatemia, which often presents during the treatment phase of DKA.^3^

Phosphate is the most abundant intracellular anion in the body and is among others involved in bone mineralization and energy metabolism.^4^ Approximately 85% of phosphate is stored in teeth and bones, merely 1% is in the extracellular compartment. In DKA, net (urinary) loss of phosphate occurs because of a transcellular shift, osmotic diuresis and reduced renal phosphate reabsorption by the Na-Pi transporters in the renal proximal tubule (due to acidosis and hyperglycemia).^5^ During insulin and fluid repletion in the treatment of DKA, phosphate shifts from the extracellular to the intracellular compartment, worsening hypophosphatemia. However, in clinical settings a normophosphatemia or even hyperphosphatemia (>1.5 mmol/L) may also be found at admission. In this phase, the transcellular shift eliminates the net urinary loss of phosphate and sometimes even exceeds. International guidelines indicate the determination of Pi during the treatment of DKA^6^ and our national guideline^7^ even states that it should be measured 1–3 times in the first 24 hours. Phosphate supplementation is recommended at levels <0.32 mmol/L.

Studies investigating the incidence, determinants and consequences of hypophosphatemia in DKA are scarce.^8–10^ A retrospective study in 43 patients with DKA^11^ showed that 63% initially presented with hyperphosphatemia, while during therapy 90% of the patients became hypophosphatemic and 11% developed a severe hypophosphatemia (phosphate concentrations <0.32 mmol/L). A relationship has been found between initial pH levels and hypophosphatemia during therapy^11^ and between insulin dose and hypophosphatemia.^12^ To date, the literature with respect to (adverse) outcomes of hypophosphatemia in DKA is limited to case reports.^13 14^

Given scarce literature on the prevalence of hypophosphatemia during DKA on one hand and the lack of evidence concerning outcomes on the other hand, we aimed to investigate the prevalence and clinical outcomes of hypophosphatemia during DKA.

## Research design and methods

### Design

This is a cohort study among individuals with T1DM admitted because of DKA. This study was executed at two hospitals in the Netherlands: Isala (Zwolle, the Netherlands, a secondary care and teaching hospital) and the University Medical Center Groningen (UMCG, located in Groningen, the Netherlands, an academic hospital).

### Outcomes

Primary outcome was the prevalence of hypophosphatemia in DKA (defined as concentrations <0.80 mmol/L) during the treatment phase of DKA. As secondary outcomes, we investigated the determinants of hypophosphatemia on clinical outcomes, such as length of hospital stay. Furthermore, we investigated the compliance with current guidelines (stating phosphate measurement in the first 24 hours of DKA and phosphate therapy should be given at levels <0.32 mmol/L).

### Patients

Patients were included in the present analysis if they were aged between 18 and 70 years and diagnosed with T1DM (defined as presence of autoantibodies and/or c-peptide concentrations).

Both patients with known T1DM as well as patients with new-onset T1DM presenting with a DKA were included. DKA was defined according to current guidelines,^6^ that is, blood glucose levels >15 mmol/L, presence of urine ketones (>2+ on urine dipsticks), pH levels <7.30 kPa, serum bicarbonate <18 mmol/L and an anion gap >10 mmol/L.

Patients with a known pre-existing condition that could have led to the presence of low phosphate concentrations, including hyperparathyroidism, Fanconi syndrome and chronic diarrhoea were per protocol excluded from the present analysis, but no patients fulfilling this were identified. Patients who were transferred from another hospital were excluded from this study. In addition, patients who left the hospital within 24 hours while the DKA had not yet been controlled were excluded from analyses.

Treatment of DKA is based on Dutch guidelines.^15^ In brief, this guideline states fluid therapy (0.9% NaCl) and insulin therapy via a continuous infusion of 0.04–0.07 IU/kg/hour, without a prior bolus. Bicarbonate therapy is to be considered when pH levels dropped below 6.9 kPa.

Intravenous therapy of potassium is considered if plasma potassium is <5.5 mmol/L.

Phosphate therapy should be considered in severe hypophosphatemia (<0.32 mmol/L).

### Data collection and measurements

For this study, data were collected using standardized forms. Due to the retrospective study design, blood samples and vital sign monitoring were not performed according to strict study protocols. Collection of data was executed by investigating medical records of patients admitted to hospital in the years 2005–2020. Specific tools, embedded in the electronic health records (EHR) software, that is, slicer dicer^16^ and CTcue,^17^ were used to identify patients. The following search terms were used: DKA in medical history or T1DM in medical history and in addition glucose levels >15 mmol/L, pH levels <7.30 kPa, serum bicarbonate <18 mmol/L.

Using this strategy, we analysed all UMCG cases (104) and we blindly and randomly selected a part of the Isala cases (28) that brought us to a total of 132 analysed cases.

Baseline data, including laboratory measurements or prehospital insulin use, were collected on hospital entry. Blood samples at arrival at the emergency department were defined as initial measurements. The assumed lowest phosphate was determined on the basis of all phosphate measurements during the treatment phase of DKA. This treatment phase was defined as 1.5 hours from admission to the end of the treatment, based on the fact that treatment usually starts after 30–60 min of arrival and that the effects of treatment can be determined after at least 3060 min.

We assessed whether there was a complication of hypophosphatemia according to hospitalization notes in the EHR. Additionally, length of hospitalization was determined by the number of hours from hospital admission until actual discharge. Length of DKA was calculated as the number of hours from the first blood test confirming DKA until the blood glucose was <15 mmol/L, and at least two of the following criteria: pH >7.30, plasma bicarbonate >15 mmol/L, anion gap within the normal ranges (8–12 mmol/L), beta-hydroxybutyrate <0.5 mmol/L.

### Statistical analysis

Q-Q plots and detrended histograms were used to define whether data are normally or not normally distributed. Descriptive summaries included the mean with SD for normally distributed variables and the median with the IQR 25th–75th percentile for non-normally distributed variables. For categorical variables, absolute number of patients (%) for phosphate is shown. The Student’s t-test was used for normally distributed variables and the Mann-Whitney U test was used for non-normally distributed variables.

Univariable analysis for correlation, using the Pearson’s product-moment correlation coefficient for continuous data and point-biserial correlation coefficient for categorical data, was performed to investigate the association between serum phosphate and other variables.

Linearity was checked on a scatter plot. Extreme outliers defined as 3× IQR from the 25th or 75th percentile were identified on a box-whisker plot were excluded. Next, multivariable linear regression analysis (method: backward-entry) was performed to investigate associations between serum phosphate as dependent variable and multiple independent variables. Variables were used in the multivariable model based on previous literature (HCO3^−^) or in case the p value was ≤0.1 in the univariable analysis. The model was checked for collinearity (variable with variance inflation factor (VIF) >10 was determined as collinear^18^) and for variables with a non-parametric distribution, natural logarithmic (ln) transformation was performed. Acid base disturbance can be measured by pH, bicarbonate and pCO2. As we anticipated that in the multivariate regression analysis these variables were collinear, we decided to only use bicarbonate in the model. The quality of the model was described using the accuracy of the variance prediction by the adjusted R^2^ value. Normality and homoscedasticity of residuals were checked with normality plots and scatter plot. For all analyses, a (two-sided) p value of <0.05 was considered statistically significant. All analyses were performed using SPSS V.25.0 (Chicago, Illinois, USA).

## Results

### Baseline characteristics

After analysis of all 132 cases, 5 cases were excluded from analyses because of leaving the hospital within 24 hours after presentation of DKA. No cases needed to be excluded based on the pre-existing conditions that could have led to low phosphate levels.

As a result, baseline characteristics of 132 episodes of DKA among 80 patients, with their first presentation at the UMCG (n=99) or Isala (n=28), were eligible for analyses and are presented in table 1.

**Table 1:** Baseline characteristics

Demographics	All (n=127)
Age (years)	28.4 (21.6–46.6)
Female gender (%)	64 (50.4)
BMI (kg/m^2^)	23.5 (4.4)
Current smoker (%)	56 (44.1)
Systolic blood pressure (mm Hg)	127 (22)
Diastolic blood pressure (mm Hg)	73 (16)
Temperature (°C)	36.5 (35.8–37.0)
Diabetes duration (years)*	13.1 (8.9–24.9)
Insulin dose (U/day)*	48 (32–72)
Use of MDI (%)*	70 (61)
Use of CSII (%)*	45 (39)
Use of sensor (%)*	12 (10)
Microvascular complications (%)*	48 (42)
Retinopathy (%)*	26 (22)
Nephropathy (%)*	29 (25)
Neuropathy (%)*	19 (17)
Macrovascular complications (%)*	21 (18)
Cause DKA	
New-onset T1DM (%)	12 (9)
Therapy non-adherence (%)	45 (35)
Infection/Inflammation (%)	33 (26)
Laboratory measurements	
Hb (mmol/L)	8.9 (1.3)
White blood cell count (109/L)	18.1 (11.6–24.9)
Platelet count (109/L)	333 (100)
CRP (mg/L)	9 (4–22)
Creatinine (μmol/L)	87 (69–117)
eGFR (mL/min/1.73 m^2^)	82.6 (40)
Sodium (mmol/L)	134 (7)
Potassium (mmol/L)	5.2 (1.0)
Glucose (mmol/L)	32(25-42)
Phosphate (mmol/L)	1.70 (1.35–2.70)
Calcium (mmol/L)	2.39 (2.26–2.47)
Urea (mmol/L)	9.2 (6.0–12.4)
Chloride (mmol/L)	93 (9)
Albumin (mmol/L)	45 (6)
LDH (U/L)	187 (159–236)
ASAT (U/L)	23 (15–31)
ALAT (U/L)	21 (14–35)
Alkaline phosphatase (U/L)	139 (58)
Gamma-GT (U/L)	25 (18–46)
Bilirubin total (μmol/L)	6 (3–11)
pH (kPa)	7.13 (7.02–7.21)
Serum bicarbonate (mmol/L)	8.3 (4.2)
Arterial pCO2 (kPa)	3.1 (1.2)
Arterial saturation (%)	97 (96–98)
Lactate (mmol/L)	3.0 (2.0–4.4)

Data are presented as number (%), mean (SD) or median (IQR).

*Excluding patients with new-onset T1DM DKA.

ALAT, alanine aminotransferase; ASAT, aspartate aminotransferase; BMI, body mass index; CRP, C reactive protein; CSII, continuous subcutaneous insulin infusion; DKA, diabetic ketoacidosis; eGFR, estimated glomerular filtration rate; EMV, Eye opening, best Motor response, best Verbal response; Gamma-GT, gamma glutamyl transferase; Hb, hemoglobin; HbA1c, glycosylated hemoglobin; LDH, lactate dehydrogenase; MDI, multiple daily injection; T1DM, type 1 diabetes mellitus.

Median age at DKA presentation was 28 (22–46) years, 45% (36/80) were female. The diabetes duration, not including new-onset T1DM DKA, was 13.2 (8.9–25.5) years with glycosylated hemoglobin levels of 93 (26.2) mmol/mol. At admission, phosphate levels were determined in 58 cases, 2 (3%) had a hypophosphatemia (<0.80 mmol/L), 18 (31%) had a normophosphatemia and 38 (66%) had a hyperphosphatemia (>1.50 mmol/L). Median phosphate concentrations at admission were 1.70 (1.35–2.70) mmol/L.

### Phosphate determinations and lowest phosphate levels

Phosphate levels were measured during the treatment phase in 66% (84/127) of all cases. Hypophosphatemia was present in 74% (62/84) and severe hypophosphatemia (<0.32 mmol/L) in 23% (19/84). The median lowest phosphate level during the treatment phase of DKA was 0.54 (0.32–0.83) mmol/L. When measured, the median number of measurements were 2 (1–4) times in the first 24 hours of admission. When hypophosphatemia occurred, the phosphate levels were measured until within normal values in 53% of all cases. The time to lowest phosphate was 16 (8–23) hours.

### New-onset T1DM DKA

Nadir phosphate levels were significantly lower in patients suffering from new-onset T1DM DKA: 0.26 (0.19–0.31) mmol/L vs 0.57 (0.45–0.88) mmol/L. The time to nadir phosphate was not significantly different between these groups. Also, pH levels were significantly lower in new-onset T1DM DKA compared with DKA among persons with known T1DM: 7.15 (7.04–7.21) kPa vs 7.03 (6.94–7.11) kPa.

### Phosphate therapy

Among the cases with severe hypophosphatemia during DKA therapy, 79% (15/19) received phosphate therapy, in 11% (2/19) it was unknown whether therapy was given and 11% (2/19) did not receive phosphate therapy. Thirty per cent (13/43) received phosphate therapy with phosphate levels between 0.32 and 0.80 mmol/L. One patient received phosphate therapy but did not develop hypophosphatemia (lowest phosphate 1.03 mmol/L). In 86% (25/29) of the cases that received phosphate therapy, phosphate levels were measured until levels >0.80 mmol/L.

In addition, 24% (6/25) of this group subsequently developed hyperphosphatemia.

### Predictors of hypophosphatemia

In univariate regression analyses, hypertension, new-onset T1DM DKA, hemoglobin (Hb) and sodium were negatively associated with lowest phosphate levels. On the other hand, creatinine, potassium, pH, bicarbonate and pCO2 were positively associated with lowest phosphate levels (table 2).

**Table 2:** Multivariate regression for lowest phosphate during the first 24 hours of treatment in DKA

	Univariate standardized beta coefficient	P value	Multivariate standardized beta coefficient	P value	Part correlation
Age (years)	0.042	0.706			
Female gender (%)	−0.055	0.622			
BMI (kg/m^2^)	−0.118	0.290			
Current smoker (yes=1)	0.118	0.289			
Smoking (pack-years)	−0.176	0.279			
Drugs (yes=1)	0.018	0.878			
Use of alcohol (U/week)	0.061	0.676			
Hypertension	−0.230	**0.038**	−0.066	0.551	
Temperature (°C)	0.006	0.964			
EMV score	0.092	0.449			
Diabetes duration (years)	0.084	0.477			
Earlier DKA (yes=1)	0.251	0.031			
Use of pen (yes=1)	0.006	0.959			
Use of pump (yes=1)	−0.006	0.959			
Use of sensor (yes=1)	−0.203	0.081			
Insulin dose (U/day)	0.159	0.322			
HbA1c (%)	−0.040	0.773			
HbA1c (mmol/mol)	−0.008	0.956			
Microvascular complications (yes=1)	0.052	0.659			
Retinopathy (yes=1)	−0.034	0.771			
Neuropathy (yes=1)	0.122	0.298			
Nephropathy (yes=1)	0.059	0.613			
Macrovascular complications (yes=1)	0.041	0.727			
Cause DKA					
New-onset T1DM (yes=1)	−0.341	**0.002**	−0.089	0.437	
Therapy non-adherence (yes=1)	0.181	0.129			
Infection/Inflammation (yes=1)	−0.053	0.660			
Laboratory measurements					
Hb (mmol/L)	−0.397	**0.000**	−0.330	0.002	−0.367
White blood cell count (10^9^/L)	−0.156	0.166			
Platelet count (10^9^/L)	−0.132	0.241			
CRP (mg/L)	−0.091	0.427			
Serum creatinine (μmol/L)	0.269	**0.016**	0.100	0.414	
eGFR (mL/min/1.73 m^2^)	−0.327	0.055			
Serum sodium (mmol/L)	−0.223	**0.046**	−0.136	0.202	
Serum potassium (mmol/L)	0.247	**0.027**	0.059	0.680	
Serum glucose (mmol/L)	0.112	0.330			
Serum phosphate (mmol/L)	0.251	0.123			
Serum urea (mmol/L)	0.254	**0.026**	0.219	0.508	
pH (kPa)	0.229	**0.057**			
Serum bicarbonate (mmol/L)	0.390	**0.001**	0.431	0.000	0.457
Arterial pCO2 (kPa)	0.392	**0.000**			
Arterial saturation (%)	−0.243	0.104			
Lactate (mmol/L)	0.056	0.651			

pH and arterial pCO2 were excluded because of collinearity with bicarbonate levels. Significant (p<0.05) outcomes are presented in bold.

BMI, body mass index; CRP, C reactive protein; DKA, diabetic ketoacidosis; eGFR, estimated glomerular filtration rate; EMV, Eye opening, best Motor response, best Verbal response; HbA1c, glycosylated hemoglobin; T1DM, type 1 diabetes mellitus.

In multivariate linear regression analysis, only baseline HCO3^−^ and Hb were independently associated with lowest phosphate levels in first 24 hours of admission.

### Clinical signs and consequences of hypophosphatemia

The hospital charts did not reveal that any patient suffered from symptoms related to hypophosphatemia. Two patients died during or shortly after admission, they had three and eight previous episodes of DKA. One patient died due to DKA, after leaving the hospital prematurely against medical advice (lowest phosphate 0.54 mmol/L). One patient died during hospitalization for DKA after being admitted with a cardiac arrest (lowest phosphate 1.03 mmol/L). Seizures and confusion, without a clear cause, was reported in another case (lowest phosphate 0.26 mmol/L).

Hypophosphatemia was not associated with intensive care unit admission, prolonged duration of DKA or hospitalization in univariate analysis. Twenty-nine cases left the hospital with hypophosphatemia, none returned to the hospital with signs of hypophosphatemia.

## Discussion

This study provides insight in the course and determinants of phosphate concentrations in DKA among adults. On admission to the hospital for DKA, 66%of all cases in which phosphate levels were measured at baseline present with hyperphosphatemia. However, during the treatment phase of DKA, hypophosphatemia occurred in 74% and a severe hypophosphatemia even in 23%. Nadir lowest phosphate level during the DKA treatment was 0.54 mmol/L, with a median duration of 16 hours to reach this phosphate level. Baseline bicarbonate and Hb levels, and thus severity of DKA, are determinants for nadir lowest phosphate levels during the treatment phase of DKA.

It has long been known that serum phosphate levels decrease during the treatment phase of DKA.^3^ In accordance with data from the retrospective study of Shen *et al*,^11^ low pH levels were associated with hypophosphatemia in our study as well. Acid-base disturbances are the main predictor for hypophosphatemia in DKA because the degree of acidosis contributes to the extracellular shift of phosphate and osmotic diuresis.

Furthermore, reduced tubular reabsorption of phosphate, as a direct effect of acidosis, and subsequent increased urinary phosphate excretion, contribute to low phosphate levels.^19^ Baseline Hb was also associated with lowest phosphate which has not been demonstrated before. Along with acid base disturbances, Hb levels represent volume depletion that occurs with DKA due to osmotic diuresis, resulting in net phosphate loss.^3^

During the treatment of DKA, rising pH levels and reversal of insulinopenia, increase intracellular glycolysis, which uses phosphate to form ATP.^20 21^ As a result of this, the intracellular amount of phosphate will decrease and in turn extracellular phosphate will enter the cell to compensate for this loss. The risk of hypophosphatemia therefore increases with increasing pH and bicarbonate levels. This also explains why the lowest phosphate develops relatively late in the treatment phase, in this study after 16 (8–23) hours, on recovery of pH. In the retrospective study by Shen *et al*^11^ among 43 patients with T1DM with 64 episodes of DKA, the prevalence of hypophosphatemia was 90% (compared with 74% in the present study) with a lowest phosphate level (0.58±0.19 mmol/L) that is almost equal to the present study. The difference in hypophosphatemia prevalence could be well explained by different phosphate measurements (due to different guidelines), or that the phosphate was determined later in the treatment phase, which explains the longer time to lowest phosphate in this comparative study (22 hours).

An important finding of the current study is that in 66% of the cases phosphate levels were determined, according to current international guideline. In 53% of the cases who developed hypophosphatemia during DKA therapy, confirmation of restoration of normal phosphate concentrations was checked. Among the patients who developed hypophosphatemia, there were no adverse events recorded in the hospital charts which were likely associated with low phosphate levels or lack of therapy. Although the limitations of this retrospective study do not allow firm conclusions, it may suggest that hypophosphatemia during DKA does not lead to serious clinical consequences. Furthermore, as previous guidelines also recommend repeated phosphate levels, it could also be concluded that guidelines are not well complied.^22^

As such, our findings question the relevance of current guidelines.^7 15^ It could be argued that (repeated) measurements of serum phosphate should only be performed in individuals presenting with severe DKA and the interval of measurement should be longer than 24 hours.

To our knowledge, this is the first two-center study that investigated the role of phosphate in DKA among a large (>100) population. Nevertheless, limitations of the present study should be acknowledged. There may be selection bias: only a minority (n=17) of the included cases were treated in the period 2005–2010. As we expect that the actual incidence of DKA in both hospitals is higher, this may indicate that we missed data in that period. A possible explanation for this omission is the introduction of electronic patient charts in this period in both hospitals. In addition, with the use of electronic searching tools we potentially miss cases. Furthermore, in this study data were collected from two centers. As presented in online supplemental table 1, patients presenting with a DKA in the UMCG had lower baseline temperature, used more often insulin injections and less often continuous subcutaneous insulin infusion, had higher baseline sodium and LDH levels and had lower baseline bilirubin and pCO2 levels. Because of the retrospective nature, there were no standardized measurement protocols for phosphate and there is missing data. As such, the lowest phosphate should be interpreted as an *assumed* lowest. As phosphate determinations were snapshots, there is a considerable chance that we missed the actual lowest phosphate and we overestimated or underestimated the time until the lowest phosphate occurred. As phosphate was no longer determined after 24 hours in almost half of the patients, the actual time to lowest phosphate could well be even later than described. It should be noted that the population studied consisted of adults. As such, the role of phosphate in DKA among children with T1DM remains to be studied. As hypertension was a significant factor in the univariate analysis, a future study may focus on the potential association between medication for hypertension and hypophosphatemia, as many of these drugs target the kidney and therefore could have an effect of renal phosphate losses.

10.1136/bmjdrc-2020-002018.supp1 Supplementary data

## Conclusions

Hypophosphatemia during the treatment phase of DKA occurs in 74% and its main determinant is the severity of acidosis. Although limitations of the retrospective nature of this study should be taken into account, it seems that no clinical symptoms or adverse effects of hypophosphatemia were present in this study. However, more studies should be performed on this topic before firm conclusions can be drawn.
