# Supplementary material for: Incidence and determinants of hypophosphatemia in diabetic ketoacidosis: an observational study
Source: BMJ Open Diabetes Res Care. 2021 Feb 17;9(1):e002018. doi: 10.1136/bmjdrc-2020-002018 (PMC7893606; doi:10.1136/bmjdrc-2020-002018)
Supplement: Supplementary data [file bmjdrc-2020-002018supp001.pdf]

Supplements

S1. Baseline characteristics of both centres.

**Demographics**	UMCG (n=99)	Isala(n=28)	p
Age [years]	28.4 [21.9-45.3]	30.7 [21.1-37.1]	.626
Female gender (%)	53 (54)	11 (39)	.183
BMI (kg/m2)	23.1 (4.2)	24.8 (5.1)	.080
Current smoker (%)	46 (46)	10 (36)	.900
Systolic Blood Pressure (mm/hg)	127 (22)	128 (22)	.826
Diastolic Blood Pressure (mm/hg)	73 (16)	75 (16)	.539
Temperature [°dfC]	36.4 [35.7-37.0]	36.7 [36.4-37.1]	**.041**
Diabetes duration [years]*	13.0 [7.4-25.9]	16.9 [10.6-23.6]	.420
Insulin dose [U/day]*	48 [32-72]	24 [23-67]	.080
Use of MDI (%)*	62 (69)	8 (32)	**.001**
Use of CSII (%)*	28 (31)	17 (68)	**.002**
Use of sensor (%)*	9 (10)	3 (13)	.878
Microvascular complications (%)*	41 (46)	7 (28)	.254
Retinopathy (%)*	22 (24)	4 (16)	.358
Nephropathy (%)*	26 (29)	3 (12)	.084
Neuropathy (%)*	15 (17)	4 (16)	.910
Macrovascular complications (%)*	16 (18)	3 (12)	.476
Cause DKA			
New onset T1DM (%)	9 (9)	3 (11)	.795
Therapy nonadherence (%)	39 (39)	6 (21)	.079
Infection/inflammation (%)	23 (23)	10 (36)	.184
**Laboratory measurements**			
Hb (mmol/L)	8.8 (1.4)	9.2 (0.9)	.055
White blood cell count [109/L]	18.6 [12.0-25.3]	16.3 [11.1-21.2]	.471
Platelet count (109/L)	331 (105)	340 (81)	.707

CRP (mg/L)	8 [4-22]	13 [5-20]	.198
Serum creatinine (μmol/L)	88 [70-124]	83 [69-112]	.695
eGFR (ml/min/1.73 m2)	79.8 (36.7)	70.0 (36.6)	.361
Serum sodium (mmol/L)	135 (7)	132 (5)	**.044**
Serum potassium (mmol/L)	5.2 (1.1)	5.2 (0.9)	.882
Serum glucose [mmol/L]	33 [25-43]	29 [25-35]	.273
Serum phosphate [mmol/L]	1.63 [1.34-2.70]	2.13 [1.36-2.97]	.477
Serum calcium [mmol/L]	2.40 [2.28-2.48]	2.36 [2.25-2.46]	.222
Serum urea [mmol/L]	9.3 [6.2-12.5]	7.6 [5.6-11.2]	.333
Serum chloride (mmol/L)	93 (8)	93 (9)	.927
Serum albumin (mmol/L)	45 (5)	44 (6)	.358
LDH [U/l]	196 [171-242]	172 [139-211]	**.005**
ASAT [U/l]	23 [15-31]	22 [16-39]	.849
ALAT [U/l]	21 [14-34]	24 [14-42]	.288
Alkaline phosphatase (U/l)	144 (59)	124 (53)	.120
Gamma-GT [U/l]	25 [19-46]	21 [14-49]	.327
Bilirubine total [μmol/l]	4 [3-10]	9 [6-12]	**.008**
pH [kPa]	7.13 [7.00-7.21]	7.16 [7.05-7.21]	.473
Serum bicarbonate (mmol/L)	8.0 (4.4)	9.2 (3.5)	.157
Arterial pCO2 (kPa)	3.0 (1.2)	3.7 (1.3)	**.015**
Arterial saturation [%]	98 [97-98]	97 [87-98]	.344
Lactate [mmol/L]	3.0 [2.0-4.4]	4.1 [2.6-4.1]	.471

BMJ Publishing Group Limited (BMJ) disclaims all liability and responsibility arising from any reliance placed on this supplemental material which has been supplied by the author(s)
